# Pathogen-Directed Antimicrobial Stewardship Audit Rounds: Evaluation of an Objectively Planned, Clinical Microbiology-Driven Antimicrobial Stewardship Approach to Influence Antimicrobial Prescriptions of Prescribers

**DOI:** 10.7759/cureus.70441

**Published:** 2024-09-29

**Authors:** Ketan Priyadarshi, D Sarumathi, Uneza Husain, Apurba S Sastry

**Affiliations:** 1 Department of Clinical Microbiology, All India Institute of Medical Sciences, Patna, Patna, IND; 2 Department of Microbiology, Jawaharlal Institute of Postgraduate Medical Education and Research, Puducherry, IND; 3 Department of Clinical Microbiology, Uttar Pradesh University of Medical Sciences, Etawah, IND

**Keywords:** antimicrobial advice, antimicrobial prescription modifications, antimicrobial stewardship, ast-guided antimicrobial therapy, bedside rounds, clinical microbiology, compliance of prescribers, culture-guided therapy, pathogen-directed therapy

## Abstract

Introduction: Correct and prompt antimicrobial prescription modifications by the treating team based on the culture and antimicrobial susceptibility test (AST) reports are crucial. Pathogen-directed antimicrobial stewardship (PD-AMS) audit rounds are a clinical microbiology-driven approach to combine prompt appropriate patient management goals with goals of preventing irrational and improper use of antimicrobials.

Methods: The study was a prospective before-after interventional study to evaluate the effectiveness of PD-AMS audit rounds in modifying antimicrobial usage by prescribers on an individual patient basis. It is divided into a two-month pre-intervention phase (no active advice is given with passive monitoring) and an intervention phase for four months, with antimicrobial therapy-related advice during PD-AMS rounds taken as “Intervention.” Antimicrobial advice was objectively classified into various categories. The PD-AMS audit round was conducted for all inpatients with a culture-proven bloodstream infection with a pathogen, and objectively classified antimicrobial advice was given through bedside rounds, telephonically, and/or written advice forms. Compliance with the advice was monitored on the next day (or at the end of 24 hours) of the PD-AMS round.

Results: The compliance with culture AST report regarding antimicrobial prescription modifications improved significantly in the intervention phase compared to the pre-intervention phase as follows: overall (83.8% (612/730) vs. 56.7% (242/427)); medicine alliance (87.4% (263/301) vs. 57.8% (115/199)); surgery alliance (87.2% (170/195) vs. 54.0% (87/161)); pediatric alliance (87.3% (138/158) vs. 66.7% (26/39)); oncology alliance (53.9% (41/76) vs. 28.6% (8/28)); critical care locations (82.0% (260/317) vs. 62.7% (89/142)); and in-patient departments (85.2% (352/413) vs. 53.7% (153/285)).

Conclusion: The clinical microbiology team should mandatorily conduct an objectively planned PD-AMS audit round to improve patient care and management and antimicrobial prescriptions through a mutual discussion with prescribers and to help address their doubts.

## Introduction

Accurate and faster culture antimicrobial susceptibility test (AST) results can improve patient care and management only if it is crucially translated into prompt and appropriate action by the treating team [1]. Guideline-recommended antimicrobial stewardship (AMS) strategies, including formulary restriction & prior authorization (FRP), prospective audit with feedback (PAF), etc., are not individualized to specific patient situations or may not occur when preliminary or final AST results are first available. The clinical microbiology team should enforce effective implementation strategies to achieve this goal [2,3].

One of the clinical microbiologist-driven AMS strategies is pathogen-directed antimicrobial stewardship (PD-AMS) audit rounds. This is also termed a culture-guided AMS audit in which the clinical microbiology team conducts ward rounds for all culture AST-positive cases and gives antimicrobial therapy and AMS-related advice [4]. It combines the prompt, appropriate patient management goals with AMS goals of preventing irrational and improper use of antimicrobials. The PD-AMS audit round would help the treating team act faster on culture AST results. It also allows the scope of mutual discussion and settlement of queries and doubts [5,6].

As it is known, “What can’t be measured, can’t be improved” or “We need to measure to improve,” the antimicrobial advice given during PD-AMS audit rounds is made objective (instead of keeping it subjective) by classifying the advice into various categories. The compliance of various prescribers to this advice can be monitored after 24-48 hours for further improvement [7].

## Materials and methods

The study was a prospective before-after interventional study conducted in a tertiary care hospital after approval by the Institutional Ethical Committee vide letter number JIP/IEC/2021/044 to evaluate the effectiveness of PD-AMS audit rounds in modifying antimicrobial usage by the treating team on an individual patient basis. The study was conducted for six months, divided into a pre-intervention phase of two months and the intervention phase of four months, with antimicrobial therapy-related AMS advice taken as “Intervention.”

The PD-AMS audit round was conducted for all inpatients with culture-proven bloodstream infection (BSI) with a pathogen during a BSI episode, which was defined as all positively flagged blood cultures (BCs) sent from the same patient during a 24-hour period as per the Clinical and Laboratory Standards Institute (CLSI) M47 document [8]. For this study, one PD-AMS round was conducted for all positively flagged BC specimens sent within 24 hours representing a single BC episode or BSI event. Subsequent BCs from the same patient having the same organism and same AST profile during a 72-hour period were excluded for a new PD-AMS round, considering it to be the same BSI event to avoid duplication [8].

PD-AMS round was conducted at two different stages for each patient’s BSI episode: preliminary PD-AMS stage (with the identification of the organism and direct bottle AST, i.e., direct susceptibility testing results in eight to 12 hours post positive flag) and final PD-AMS stage (with colony AST results). Those cases where patients had left against medical advice, expired, or were untraceable during the PD-AMS audit round were also excluded. The unit of analysis was antimicrobial advice (Table 1). Multiple antimicrobial advice to the same patient during a single BSI episode was also included as a separate unit. Compliance with advice was followed after 24 hours or the next day of giving PD-AMS advice.

**Table 1:** Categorization of antimicrobial therapy modification advice to make them objective and measurable. MIC: minimum inhibitory concentration. Adapted from Sastry AS, Priyadarshi K, Sarumathi D. 2023. Essentials of Antimicrobial Stewardship. 1st ed. Jaypee, pp. 46-56 [7].

Antimicrobial advice
(1) Empirically correct
Continue the current antimicrobials
(2) Empirically incorrect
2a. Start antimicrobials
2b. Change of spectrum (e.g. Gram-positive spectrum, Gram-negative spectrum, and antifungals)
2c. Change within spectrum
2c (i). Escalation
2c (ii). De-escalation
2c (ii A). Narrowing of the spectrum
2c (ii B). Remove overlapping spectrum
2c (ii C). Remove redundant antimicrobials
2c (ii D). Switch from intravenous to oral
2c (ii E). Stop antimicrobials
2c (iii). Change of antimicrobials
2c (iii A). Within line à for the following reasons: covers >1 organism; better breakpoint-to-MIC quotient (BMQ); resistant to the agent; allergic to the agent; not site-specific; not age-specific; organ-specific restrictions (e.g., nephrotoxic); others
2c (iii B) Across the line à for the following reasons: covers >1 organism; better breakpoint-to-MIC quotient (BMQ); resistant to the agent; allergic to the agent; not site-specific; not age-specific; organ-specific restrictions (e.g., nephrotoxic); others
(3) Administrative advice
Loading dose advice, infusion-related advice, and dosage-related advice

During the pre-intervention period, PD-AMS audit rounds were conducted, but no active advice was given to the treating team. The antimicrobial therapy-related modifications done by the treating team themselves were recorded from the patient’s case sheet on the next day (or end of ~24 hours) of authorization of the culture AST report and then categorized as per the various advice categories and subsequently compliance was calculated based on the expected advice that might have been given (Table 1).

**Figure 1:**
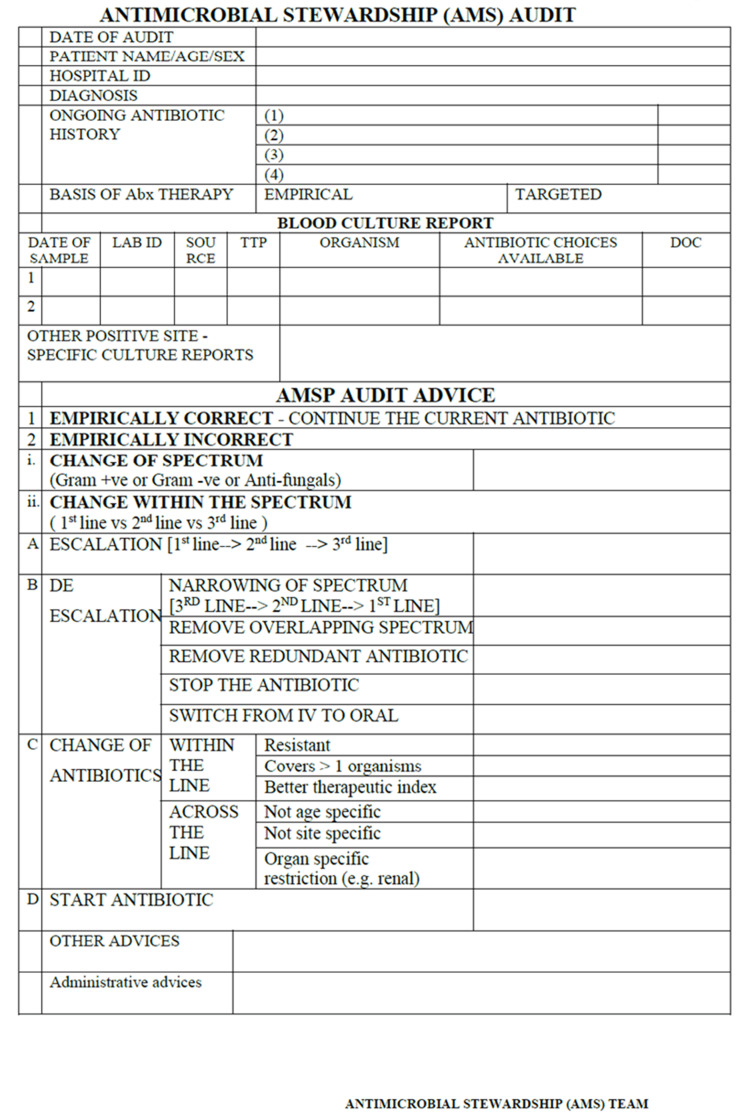
Antimicrobial advice form used during PD-AMS audit round. PD-AMS: pathogen-directed antimicrobial stewardship.

During the intervention phase, the antimicrobial advice was communicated to the treating team as a part of the PD-AMS audit round by any combination of these three methods: (1) verbal communication over the phone or through messages (contact details of the prescriber was a pre-requisite); (2) verbal communication in person at patient’s bedside during PD-AMS audit round (knowledge of patient’s location & finding treating team at bedside was a pre-requisite); (3) written communication (through PD-AMS advice form) attached to the patient’s case sheet (knowledge of patient’s location was a pre-requisite) (Figure 1). The preferred hierarchy of communication was “In person bedside round + written advice form,” followed by “telephonic communication + written advice form,” followed by “written advice form only.” Compliance with the advice was monitored on the next day (or at the end of 24 hours) of the PD-AMS round.

The antimicrobial advice that was not implemented within 24 hours of advice was considered “Non-compliance”; subsequently, the reason for non-compliance was tried to be found. Compliance was also calculated for various categories: patient care specialties (i.e., medicine alliance, surgery alliance, pediatrics alliance, and oncology-transplant alliance), patient care locations (i.e., critical care locations/ICUs or in-patient departments/IPDs), and professional cadre of the treating team (i.e., senior residents or junior residents).

The compliance with each of the PD-AMS advice during the intervention phase was compared with that in the pre-intervention phase, and the statistical significance of the change in compliance was calculated using the chi-squared test.

## Results

During the pre-intervention phase, a total of 427 antimicrobial advice were given for a total of 275 patients and 427 BSI episodes (i.e., positively flagged BCs). During the intervention phase, a total of 730 antimicrobial advice were given for a total of 394 patients and 542 BSI episodes (i.e., positively flagged BCs) (Table 2).

**Table 2:** Baseline data of the pre-intervention and intervention phase of the study. PD-AMS: pathogen-directed antimicrobial stewardship; BSI: bloodstream infection.

	Pre-intervention phase		Intervention phase	
Duration	2 months		4 months	
No. of antimicrobial advice	427		730	
No. of patients	394		275	
No. of BSI episodes	427		542	
Patient care specialty type				
Medicine alliance	199	46.6%	301	41.2%
Oncology alliance	28	6.6%	76	10.4%
Pediatrics alliance	39	9.1%	158	21.6%
Surgery alliance	161	37.7%	195	26.7%
Patient care location type				
Critical care locations (ICUs)	142	33.3%	317	43.4%
In-patient locations (IPDs)	285	66.7%	413	56.6%
Profession cadre for advice				
Junior resident (JR)	NA	NA	463	63.4%
Senior resident (SR)	NA	NA	267	36.6%
Advice mode				
Telephonically + advice form	NA	NA	159	21.8%
PD-AMS rounds + advice form	NA	NA	571	78.2%

The compliance with the AST report as a result of intervention undertaken in the form of antimicrobial advice during the PD-AMS audit round improved significantly (p-valve <0.05) in the intervention phase compared to the pre-intervention phase. The overall baseline compliance to AST reports showed approximately 27% improvement, i.e., 83.8% (612/730) in the intervention phase vs. 56.7% (242/427) in the pre-intervention phase. Similarly, improvements were seen in different treating specialties: medicine alliance = 87.4% (263/301) in the intervention phase vs. 57.8% (115/199) in the pre-intervention phase; surgery alliance = 87.2% (170/195) in the intervention phase vs. 54% (87/161) in the pre-intervention phase; pediatric alliance = 87.3% (138/158) in the intervention phase vs. 66.7% (26/39) in the pre-intervention phase. Even if there was a considerable and significant increase (p-valve <0.05) in compliance in the oncology alliance, 53.9% (41/76) in the intervention phase vs. 28.6% (8/28) in the pre-intervention phase, the overall compliance is still poor compared to other clinical services.

**Figure 2:**
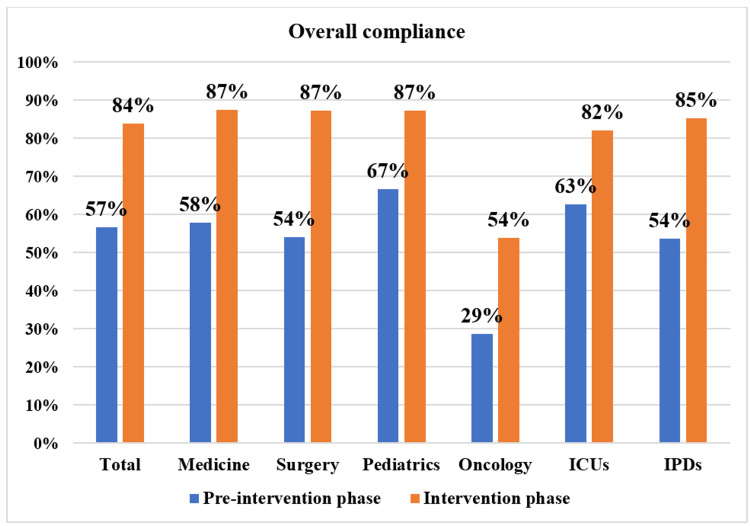
Overall compliance with PD-AMS advice in pre-intervention and intervention phases. PD-AMS: pathogen-directed antimicrobial stewardship; ICUs: critical/intensive care units; IPDs: in-patient departments.

The baseline compliance was almost similar among critical care locations (62.7%, 89/142) and in-patient departments (53.7%, 153/285) in the pre-intervention phase. The overall compliance improved significantly (p-valve <0.05) in both critical care locations, 82.0% (260/317) in the intervention phase vs. 62.7% (89/142) in the pre-intervention phase, and in-patient departments, 85.2% (352/413) in the intervention phase vs. 53.7% (153/285) in the pre-intervention phase, on the PD-AMS round. The improvement was seen slightly more in IPDs than in ICUs, but the difference was insignificant (Figure 2).

**Figure 3:**
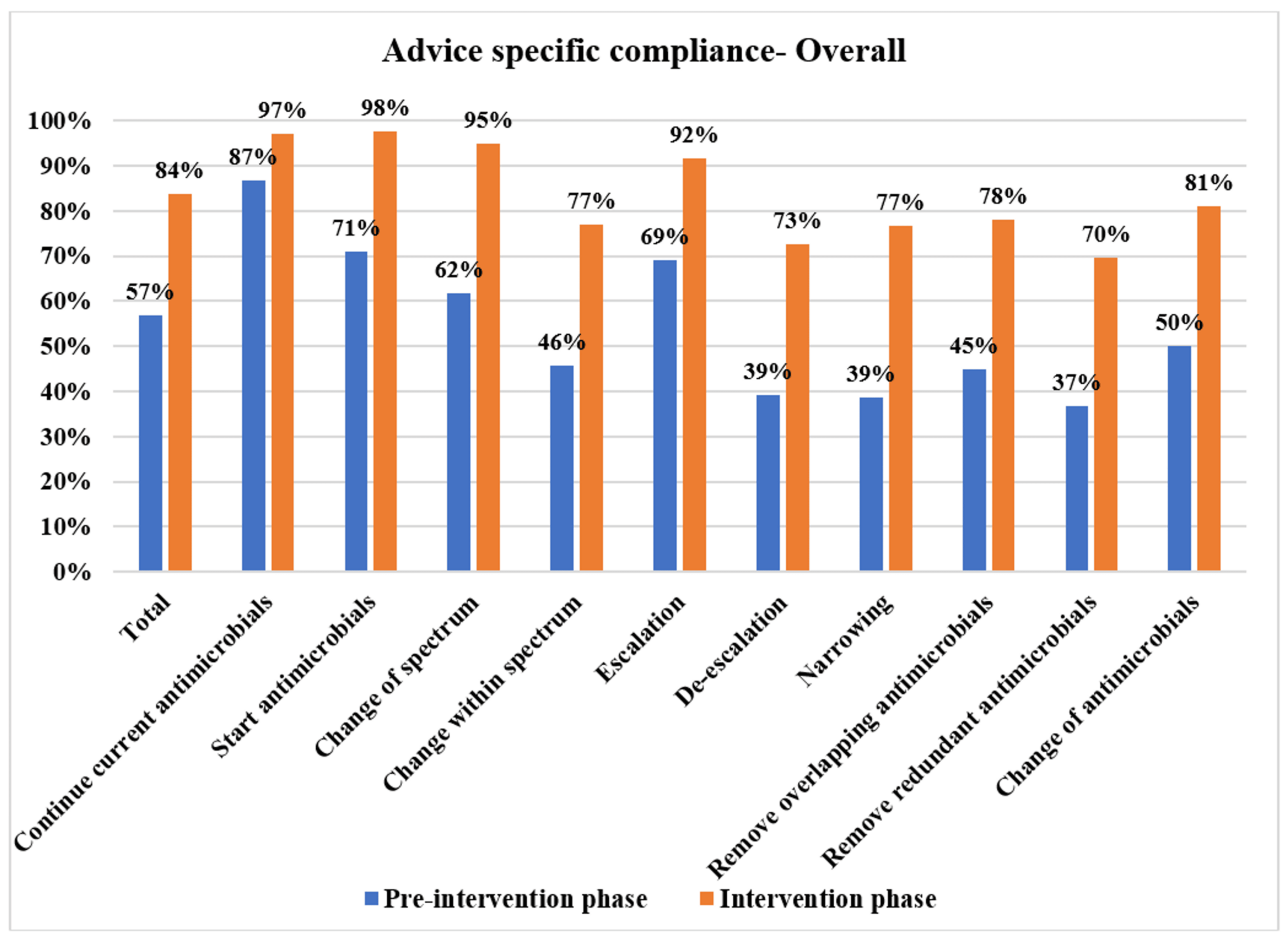
Overall compliance with various PD-AMS advice categories in pre-intervention and intervention phases. PD-AMS: pathogen-directed antimicrobial stewardship.

**Figure 4:**
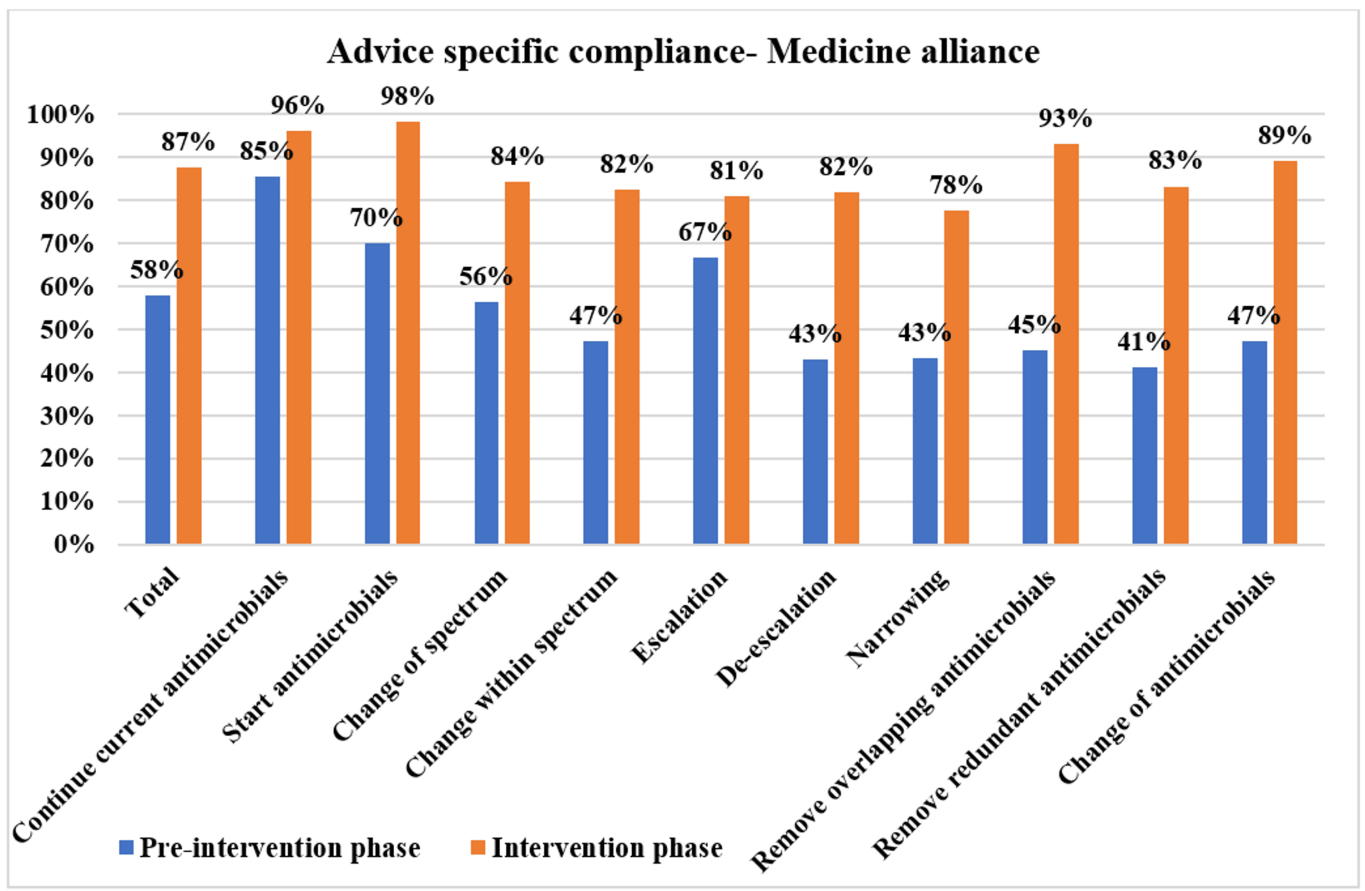
Compliance with various PD-AMS advice categories in the medicine alliance departments during pre-intervention and intervention phases. PD-AMS: pathogen-directed antimicrobial stewardship.

**Figure 5:**
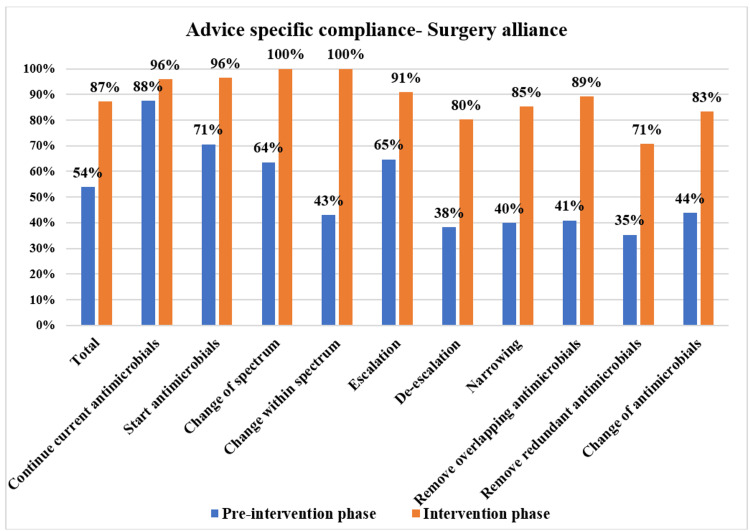
Compliance with various PD-AMS advice categories in the surgery alliance departments during pre-intervention and intervention phases. PD-AMS: pathogen-directed antimicrobial stewardship.

**Figure 6:**
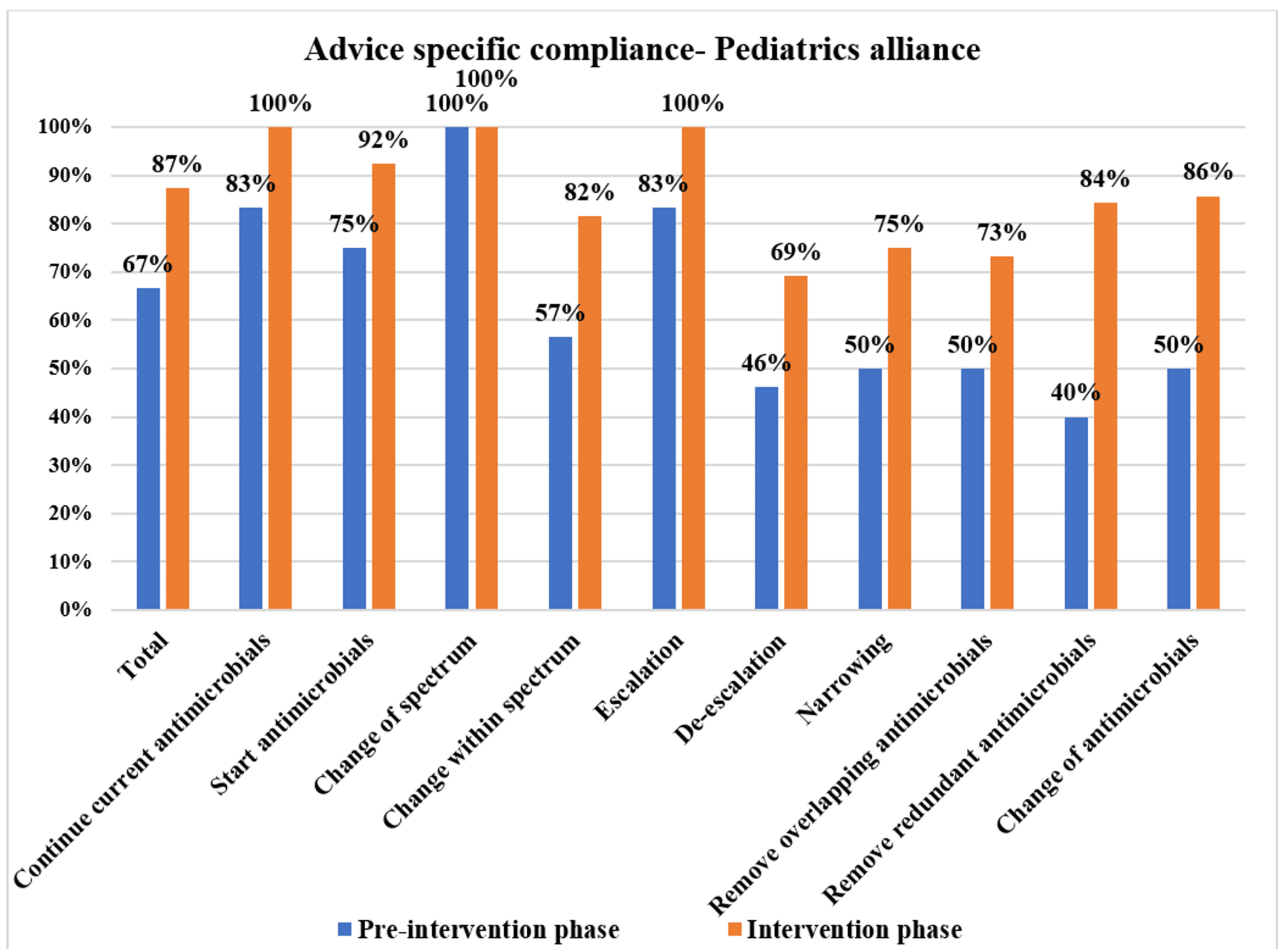
Compliance with various PD-AMS advice categories in the pediatrics alliance departments during pre-intervention and intervention phases. PD-AMS: pathogen-directed antimicrobial stewardship.

**Figure 7:**
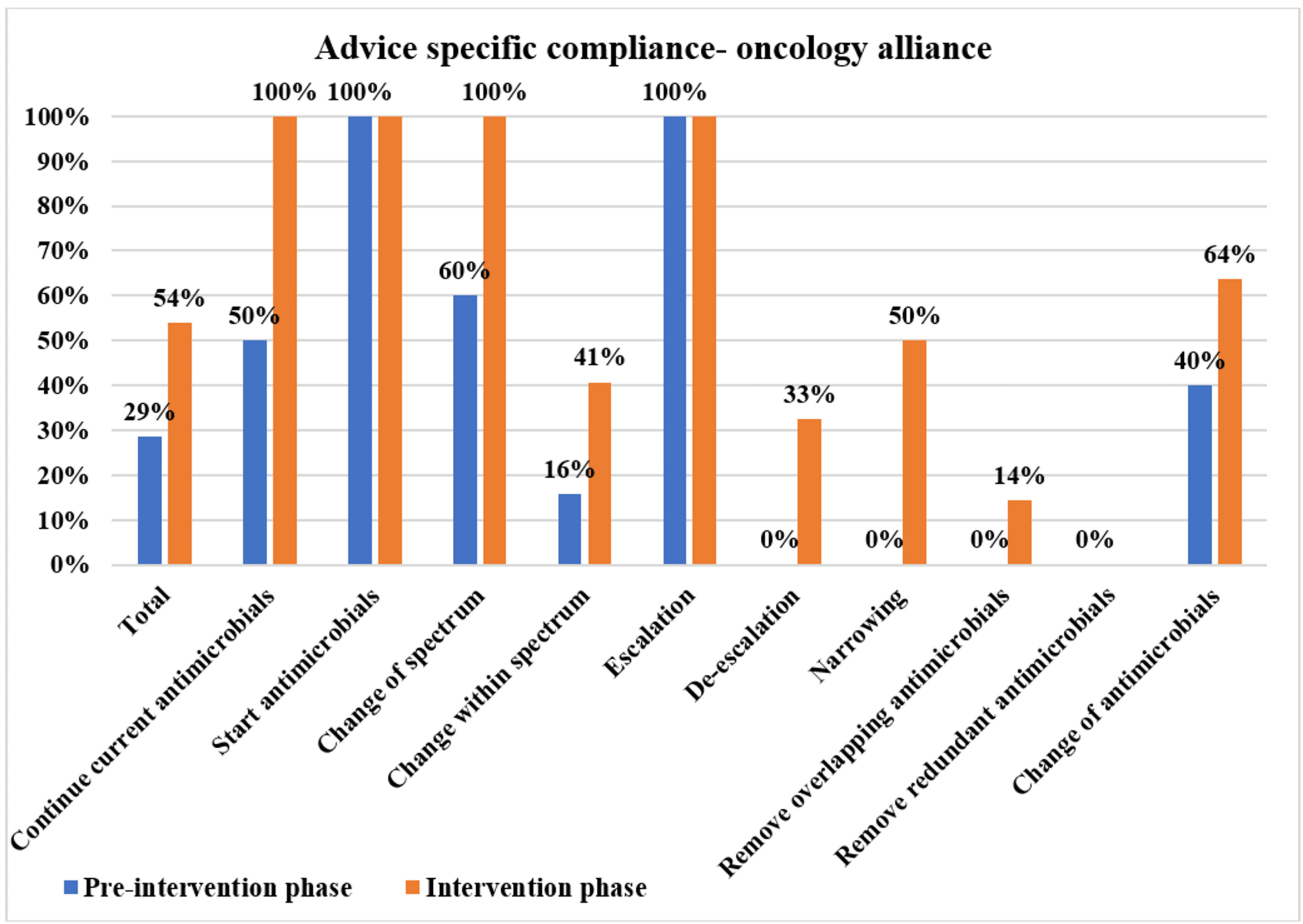
Compliance with various PD-AMS advice categories in the oncology alliance departments during pre-intervention and intervention phases PD-AMS: pathogen-directed antimicrobial stewardship.

**Figure 8:**
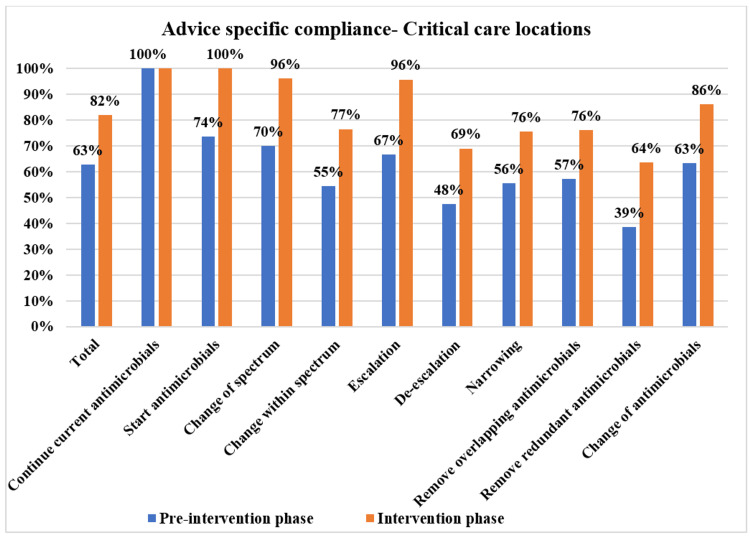
Compliance with various PD-AMS advice categories in the critical care locations during pre-intervention and intervention phases. PD-AMS: pathogen-directed antimicrobial stewardship.

**Figure 9:**
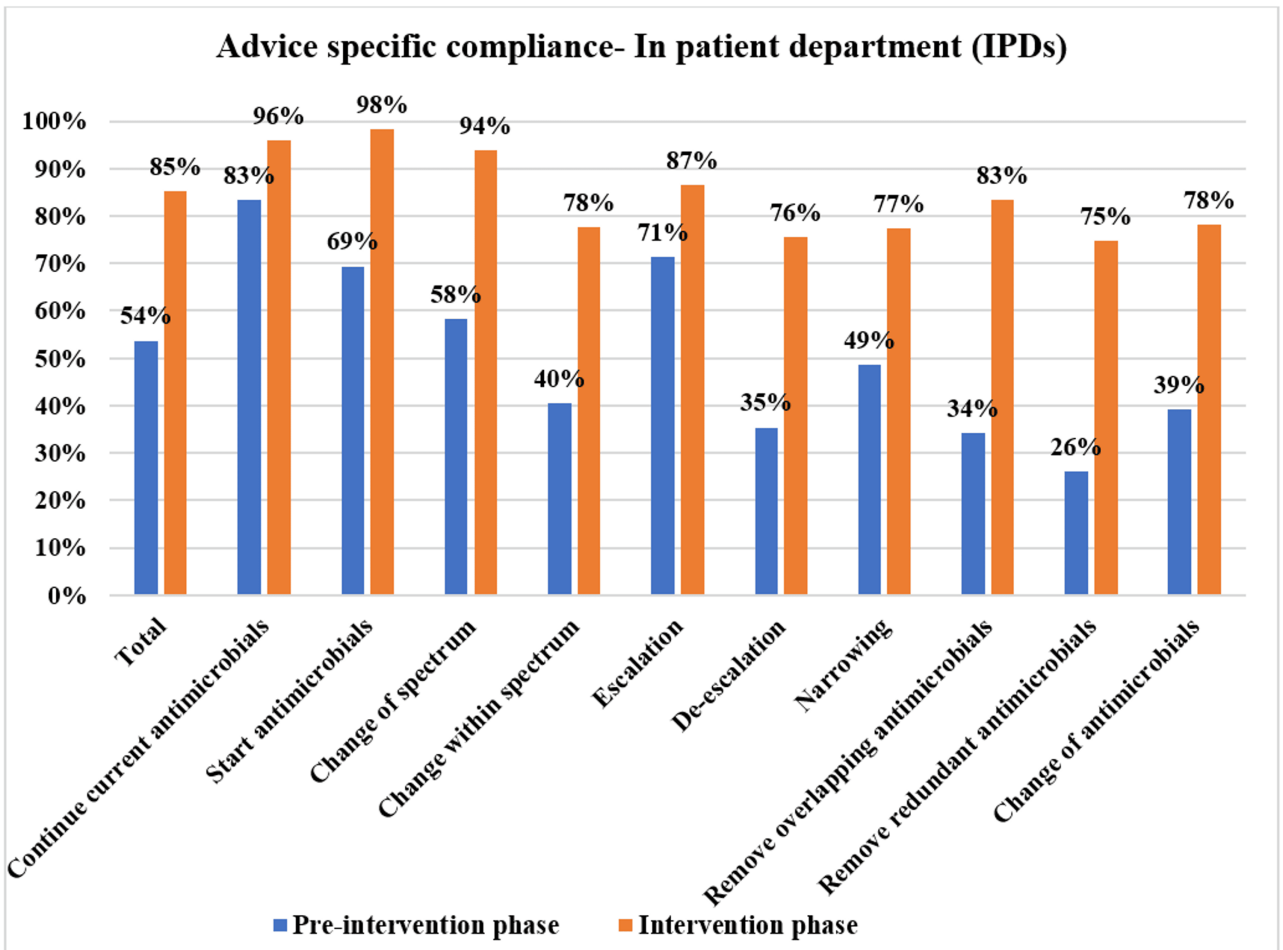
Compliance with various PD-AMS advice categories in the IPD locations during pre-intervention and intervention phases. PD-AMS: pathogen-directed antimicrobial stewardship.

**Figure 10:**
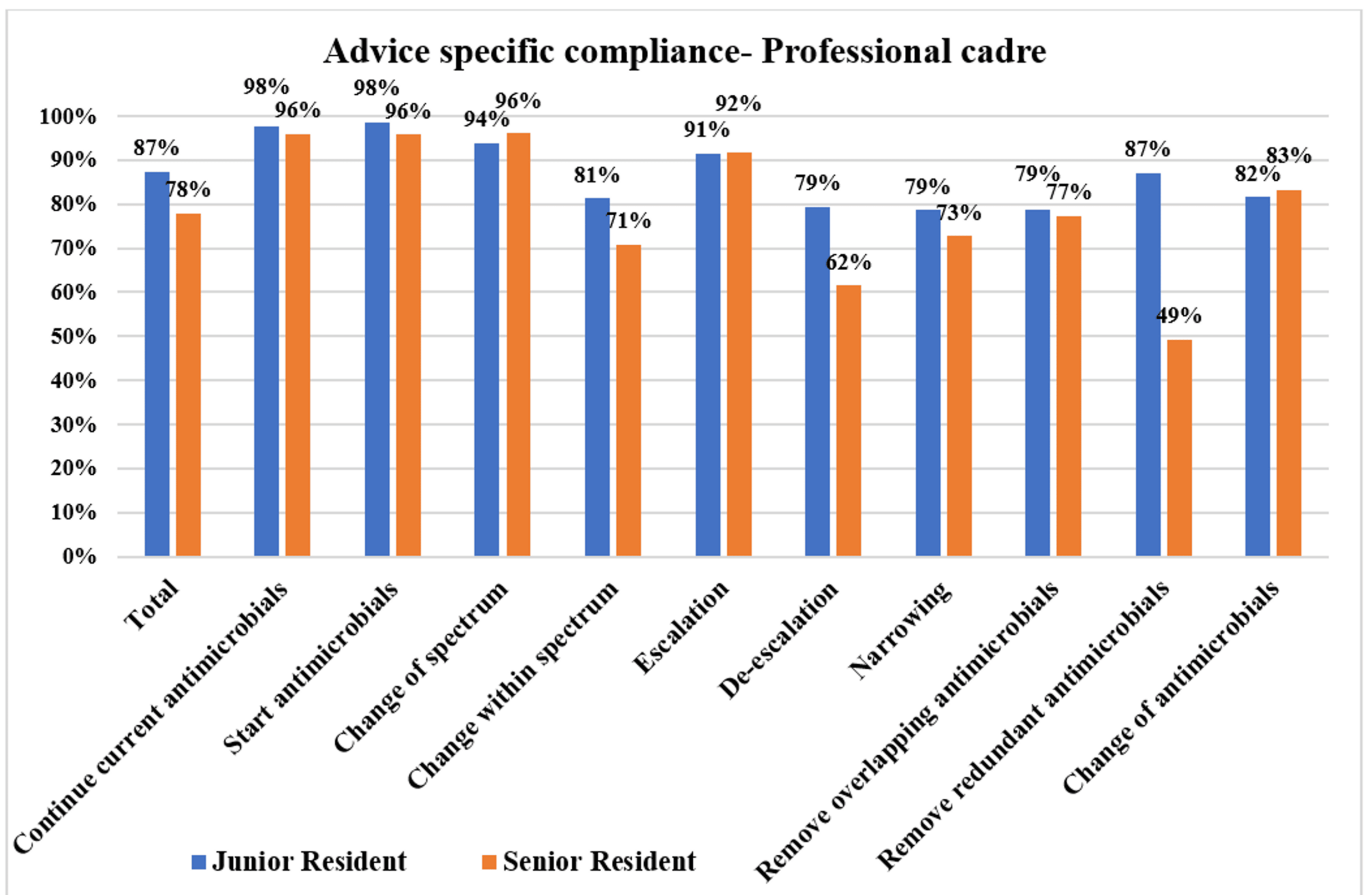
Compliance with various PD-AMS advice categories in the various professional cadres during pre-intervention and intervention phases. PD-AMS: pathogen-directed antimicrobial stewardship.

The overall PD-AMS advice category-specific compliance in the pre-intervention and intervention phases is depicted in Figure 3. The patient care specialties-specific compliance in the pre-intervention and intervention phases is depicted in Figures 4-7. The patient care location-specific compliance in the pre-intervention and intervention phases is depicted in Figures 8, 9. The professional cadre-specific compliance in the pre-intervention and intervention phases is depicted in Figure 10.

The total non-compliance found to the antimicrobial advice during the PD-AMS round in the intervention phase was 16.2% (118/612). Among various clinical services, the non-compliance was as follows: medicine alliance = 12.6% (38/301), surgery alliance = 12.8% (25/195), pediatric alliance = 12.7% (20/158), and highest among all for the oncology alliance = 46.1% (35/76).

Among the various reasons quoted by the clinical team for the non-compliance to the antimicrobial advice, these can be listed in the decreasing order as follows: (i) the patient is sick and hemodynamically unstable (50.8%, 60/118), so the clinical team was willing to continue with all the ongoing antimicrobials; (ii) the patient is moderate to severely immunocompromised (11.0%, 13/118), so the clinical team was willing to continue with all the ongoing antimicrobials; (iii) the patient was stable and doing fine without antimicrobials at present (9.3%, 11/118); (iv) suspected co-existing alternate infections clinically or source of infection pending microbiological confirmation (7.6%, 9/118); (v) parenteral formulation of antimicrobial agent was not available in the pharmacy and the patient could not be kept on oral formulation (6.8%, 8/118); (vi) the patient was improving with the ongoing antimicrobial agents and the clinical team was not willing to change (5.9%, 7/118); (vii) suspected fungal infective etiology or prophylactic antifungals (4.2%, 5/118); (viii) the patient was febrile/symptomatic or not improving with single or ongoing antimicrobial agents so more than one antimicrobial of overlapping spectrum was added (3.4%, 4/118); and (ix) higher class of antimicrobial agent better at the specific body site of infection (0.8%, 1/118). In the oncology alliance clinical services, the majority of the non-compliance was due to these three reasons: (i) the patient is sick and hemodynamically unstable (51.4%, 18/35); (ii) the patient is moderately to severely immunocompromised (34.3%, 12/35); and (iii) the patient was improving with the ongoing antimicrobial agents (11.4%, 4/35).

## Discussion

Time spent performing the diagnostic test and dispensing the reports forms a crucial factor in appropriate patient management. This is particularly important for clinical microbiological diagnostic tests like blood culture, which plays a critical role in the diagnosis of life-threatening infectious conditions like BSIs and sepsis [1,2]. However, prompt and appropriate clinical action is required to translate these faster test results into improved patient care. PD-AMS audit rounds are a modification of the prospective audit and feedback method of antimicrobial surveillance, which deals with two AMS goals simultaneously: (i) improving the effectiveness of “diagnostic stewardship” by reducing the action time of the clinical team to bring out the antimicrobial prescription modifications based on AST report; (ii) preventing the indiscriminate use of antimicrobials by bringing the change on a case-to-case basis [3-6].

In the pre-intervention phase, antimicrobial advice was not given, but compliance was still calculated based on expected antimicrobial therapy modifications. This was performed to evaluate how frequently the prescribers appropriately modify their antimicrobial prescriptions by themselves after getting the culture AST report. In the intervention phase, almost 4/5th of the antimicrobial advice was given bedside personally during the PD-AMS audit round, with only 1/5th of the advice given telephonically. In all the cases, the PD-AMS advice form was attached to the patient’s case sheet for further reference. Somehow, all the advice was given to residents and none to the faculties/consultants [4-6,9].

It was worth mentioning that the antimicrobial advice was not blindly based on one BC report. Any other previous BC reports and other clinical specimen culture AST reports for the previous five days in the hospital information system (HIS) available on the day of the PD-AMS round were taken into consideration. However, any AST report authorized after the PD-AMSP round was not taken into consideration on the day of follow-up. Antibiotics were defined into three lines, i.e., 1st line, 2nd line, and 3rd line, based on the principles of cascade reporting, and the same had been used to categorize antimicrobial advice into various types [7,10,11].

The baseline compliance with culture AST reports was found to be 56.7% after 24 hours of authorization of reports, which meant only about half of the treating prescribers had modified their antimicrobial prescription on their own as per the advice in the AST report post 24 hours, which was definitely low and a matter of concern. This compliance was slightly better in pediatric services, with compliance being almost 2/3rd (66.7%), but worst in the oncology service, with compliance being even less than 1/3rd (28.6%) [10-12].

In the pre-intervention phase, overall compliance was better for antimicrobial advice such as (i) “Continuation of ongoing antimicrobials” (86.8%). However, it was worth noting that even 13.2% of antimicrobial prescriptions were changed after the available AST reports. (ii) “Start the antimicrobials” (70.9%) and (iii) “Escalation of ongoing antimicrobials” (69.0%). This is primarily because, in this advice, either a continuation or addition of antimicrobials was advised, which is often not a major concern for prescribers and hence implemented well on their own. Overall compliance was very poor for the “De-escalation” advice (39.2%) and its various sub-types such as (i) “Narrowing” (38.6%), (ii) “Removing overlapping spectrum” (44.9%), and (iii) “Removing redundant antimicrobials” (36.8%). Overall compliance was also reasonably poor for advice such as (i) “Change of spectrum” (61.8%) and (ii) “Change of antimicrobials within the same spectrum” for reasons other than escalation and de-escalation either within the line or across the line (50%). It has been observed that any advice that involved reducing the antimicrobials prescribed had a relatively poor acceptance on their own. Similar trends in compliance with specific types of antimicrobial advice were seen in various clinical services (i.e., medicine, surgery, pediatrics, and oncology) as well as patient care locations (i.e., ICUs and in-patient departments) in the pre-intervention phase [4-6,12].

In the intervention phase, when specific antimicrobial advice was conveyed to the clinical residents through bedside round discussions and/or telephonic discussions along with advice forms, there was a significant improvement in overall compliance with the antimicrobial advice throughout the healthcare facility, which necessitates the need of mutual discussion among treating prescribers and the clinical microbiologists to improve patient management [4-6,12].

Among the clinical services, in the medicine alliance services, the lowest compliance was found in “Narrowing of the spectrum” (77.6% (45/58) in the intervention phase vs. 43.3% (13/30) in the pre-intervention phase). In the surgery alliance, the lowest compliance was found in “Removal of redundant antimicrobials” (70.7% in the intervention phase vs. 35.3% in the pre-intervention phase). In the pediatrics alliance, the lowest compliance was found in “Narrowing of the spectrum” (75% in the intervention phase vs. 50% in the pre-intervention phase) and “Removal of the overlapping spectrum” (73.3% in the intervention phase vs. 50% in the pre-intervention phase). In the oncology alliance, in the intervention phase, even after PD-AMS rounds, the lowest compliance was found in almost all the “De-escalation” advice (32.6%), which were as follows: “Removal of redundant antimicrobials” (0%), “Removal of overlapping spectrum” (14.3%), and “Narrowing of the spectrum” (50.0%). In the pre-intervention phase, compliance with all the “De-escalation” advice was 0.0%, which improved to some extent to 32.6% in the intervention phase. Almost all of the non-compliance was found in hematological malignancy patients with febrile neutropenia and low absolute neutrophil count. Compliance was better in solid organ malignancy patients, as seen in several other studies [10-12].

In the critical care locations, in the intervention phase, the lowest compliance was found in almost all the “De-escalation” advice (69.0% in the intervention phase vs. 47.5% in the pre-intervention phase), which were as follows: “Removal of redundant antimicrobials” (63.5% in the intervention phase vs. 38.5% in the pre-intervention phase); “Removal of the overlapping spectrum” (76.1% in the intervention phase vs. 57.1% in the pre-intervention phase); and “Narrowing of the spectrum” (75.6% in the intervention phase vs. 55.6% in the pre-intervention phase). Poor compliance was primarily observed in advice that involved reducing the antimicrobial therapy of patients, as the prescribers had their concerns in doing so.

In the inpatient locations, in the intervention phase, the lowest compliance was found in “Removal of redundant antimicrobials” (63.5% in the intervention phase vs. 38.5% in the pre-intervention phase), “Removal of the overlapping spectrum” (74.7% in the intervention phase vs. 26.0% in the pre-intervention phase), and “Narrowing of the spectrum” (77.4% in the intervention phase vs. 48.7% in the pre-intervention phase). Even in wards where patients were relatively less sick and usually stable, relatively poor compliance was observed in advice that involved reducing the antimicrobial therapy, as the prescribers had their concerns about doing so. In the intervention phase, compliance-wise, both the professional cadres, namely, junior residents (87.3%) and senior residents (77.9%), were similar, with no statistically significant minor differences. Senior residents (61.5%, 80/130) were found to be more non-compliant with “de-escalation” advice as compared to the junior residents (79.4%, 162/204). However, the acceptance of senior faculties or consultants could not be evaluated in this study, as almost all of the advice was given to residents during bedside rounds. This might be attributed to the highly variable time of conduction of clinical rounds by senior faculties [10-12].

In total, during the intervention phase, as compared to the pre-intervention phase, the “Non-compliance” to antimicrobial advice during the PD-AMS round was 16.2% (118/730) vs. 43.3% (185/427), which showed a significant decrease (p-value <0.05). Overall, the most common reason stated for “Non-compliance” was patients being sick and/or hemodynamically unstable (50.8%, 60/118), which was the most common reason for all clinical services and patient care locations. The common reasons for “Non-compliance” to “De-escalation” advice were patients being sick and/or hemodynamically unstable, moderate to severely immunocompromised, patients improving with ongoing higher-line antimicrobials, suspected co-existing infections, and unavailability of parenteral formulations of advised antimicrobials. The common reasons for non-compliance with “escalation,” “start antimicrobials,” or “change of antimicrobials” advice were that the patient was doing fine or was clinically stable at the time of the PD-AMS round, so the clinical team was not willing to do any antimicrobial modifications [4-6,9].

One of the limitations of the present study was that the patients with BSI were followed only for 24 hours for their antimicrobial prescriptions. Subsequent follow-up or monitoring of the antimicrobial prescription modifications was not followed. Another limitation of the study was that those patients who did not have any culture-positive BSI were not followed for antimicrobial prescriptions. This was an inherent limitation of PD-AMS audit rounds as compared to PAF rounds [13]. Still, PD-AMS rounds might help to review antimicrobial prescriptions of a larger and critical group of culture-positive cases in which implementing targeted therapy-based antimicrobial modifications is an important low-hanging fruit, with less intensive human resource involvement as compared to PAF rounds [14].

## Conclusions

Implementing a simple clinical microbiology-driven pathogen-directed culture-based audit round helps address the doubts of the treating team, and this mutual discussion helps them bring appropriate and prompt modifications to the antimicrobial prescriptions based on the AST report. Objectively classified antimicrobial advice makes the audit measurable and, hence, improvable. Thus, objectively planned PD-AMS audit rounds should be mandatorily conducted by the clinical microbiology team to improve patient care and management.
